# α2β1 Integrin, GPVI Receptor, and Common FcRγ Chain on Mouse Platelets Mediate Distinct Responses to Collagen in Models of Thrombosis

**DOI:** 10.1371/journal.pone.0114035

**Published:** 2014-11-21

**Authors:** Robin J. Marjoram, Zhengzhi Li, Li He, Douglas M. Tollefsen, Thomas J. Kunicki, S. Kent Dickeson, Samuel A. Santoro, Mary M. Zutter

**Affiliations:** 1 Department of Pathology, Microbiology and Immunology, Vanderbilt University Medical Center, Nashville, TN, United States of America; 2 Children's Hospital of Orange County, Orange, CA, United States of America; 3 Department of Medicine, Washington University School of Medicine, St. Louis, MO, United States of America; Chang Gung University, Taiwan

## Abstract

**Objective:**

Platelets express the α2β1 integrin and the glycoprotein VI (GPVI)/FcRγ complex, both collagen receptors. Understanding platelet-collagen receptor function has been enhanced through use of genetically modified mouse models. Previous studies of GPVI/FcRγ-mediated collagen-induced platelet activation were perfomed with mice in which the FcRγ subunit was genetically deleted (FcRγ^−/−^) or the complex was depleted. The development of α2β1^−/−^ and GPVI^−/−^ mice permits side-by-side comparison to address contributions of these collagen receptors *in vivo* and *in vitro*.

**Approach and Results:**

To understand the different roles played by the α2β1 integrin, the GPVI receptor or FcRγ subunit in collagen-stimulated hemostasis and thrombosis, we compared α2β1^−/−^, FcRγ^−/−^, and GPVI^−/−^ mice in models of endothelial injury and intravascular thrombosis *in vivo* and their platelets in collagen-stimulated activation *in vitro*. We demonstrate that both the α2β1 integrin and the GPVI receptor, but not the FcRγ subunit influence carotid artery occlusion *in vivo*. In contrast, the GPVI receptor and the FcRγ chain, but not the α2β1 integrin, play similar roles in intravascular thrombosis in response to soluble Type I collagen. FcRγ^−/−^ platelets showed less attenuation of tyrosine phosphorylation of several proteins including RhoGDI when compared to GPVI^−/−^ and wild type platelets. The difference between FcRγ^−/−^ and GPVI^−/−^ platelet phosphotyrosine levels correlated with the *in vivo* thrombosis findings.

**Conclusion:**

Our data demonstrate that genetic deletion of GPVI receptor, FcRγ chain, or the α2β1 integrin changes the thrombotic potentials of these platelets to collagen dependent on the stimulus mechanism. The data suggest that the FcRγ chain may provide a dominant negative effect through modulating signaling pathways in platelets involving several tyrosine phosphorylated proteins such as RhoGDI. In addition, these findings suggest a more complex signaling network downstream of the platelet collagen receptors than previously appreciated.

## Introduction

Hemostasis relies on the highly regulated balance of prothrombotic and antithrombotic components to prevent blood loss from the vasculature while at the same time maintaining blood fluidity. Platelets play a central role in this balance especially during arterial hemostasis and pathological thrombosis. Fibrillar collagens represent a potent prothrombotic stimulus for platelets at sites of vascular injury.

Platelets express two receptors, α2β1 integrin and the glycoprotein VI (GPVI)/Fc receptor-gamma (FcRγ) complex, that together mediate platelet adhesion and activation in response to collagens [1]–[3]. The α2β1 integrin, a heterodimeric transmembrane receptor, provides strong adhesion. GPVI, a single span transmembrane receptor with two immunoglobulin domains non-covalently associates with the FcRγ chain that contains the immunoreceptor tyrosine-based activation motif (ITAM), which in complex form the primary collagen signaling receptor [4]–[6].

A role for α2β1 integrin-mediated adhesion in vascular disease was suggested by epidemiologic studies that linked α2β1 integrin density to pathologic thrombosis and enhanced bleeding. Kunicki *et al*. demonstrated that α2β1 integrin density on the platelet surface directly correlates with platelet adhesiveness to Type I collagen [7]. We previously reported that the α2β1 integrin-deficient (α2β1^−/−^) mice clearly exhibit impaired adhesion to collagen substrates under arterial flow conditions and observed a marked decrement in thrombus formation *in vivo* following arterial injury [8].

Similarly, the importance of the GPVI/FcRγ complex in normal hemostasis was demonstrated in patients with a mild bleeding diathesis associated with either mutations in the *Gp6* gene or presence of anti-GPVI antibodies [1], [9]–[11]. Collagen or collagen-related peptides (CRPs) binding to the GPVI subunits stimulate clustering of the GPVI/FcRγ complex, tyrosine phosphorylation of the ITAM motifs within FcRγ chains, and activation of the Src family tyrosine kinases Fyn and Lyn that trigger platelet activation [12], [13]. Phosphorylation of the ITAM domain also results in activation of Syk and downstream effectors, including PLCγ2, PI3K, and small GTPases that contribute to platelet activation and aggregation [13]. Earlier studies of GPVI/FcRγ-mediated collagen-induced platelet activation and thrombus formation were carried out using mice in which either the FcRγ subunit was genetically deleted [FcRγ-deficient (FcRγ^−/−^) mice] or the complex was depleted by antibody-mediated internalization. Of note, platelets derived from FcRγ^−/−^ mice fail to express GPVI. In these studies, lack of FcRγ through genetic knockout or antibody-depletion resulted in attenuated collagen-stimulated platelet activation and thrombus formation under flow conditions *in vitro*, however the phenotype *in vivo* still remains unclear [14]–[18]. Importantly, the FcRγ^−/−^ animals also lack FcεRγI, FcγRIII, and FcγRI, and are immunodeficient with abnormalities in macrophage, NK cell, mast cell and B cell function. More recently, GPVI-deficient (GPVI^−/−^) mice were developed. These mice were reported to be viable and fertile, and to exhibit normal bleeding times. However, GPVI^−/−^ platelets did not aggregate in response to collagen or GPVI-specific collagen related peptide (CRP) [15]. Although GPVI-null platelets did not form aggregates when perfused over a collagen surface, they did form an adherent monolayer. Kato *et al*. attributed the residual adhesion solely to von Willebrand factor [15]. Conversely, other groups showed decreased platelet adhesion to collagen upon loss of GPVI [14], [18], [19]. These discrepancies suggest that much remains to be understood about the mechanisms of collagen receptor signaling in platelets.

The development of α2β1^−/−^, FcRγ^−/−^, and GPVI^−/−^ mice allowed us to compare the roles and contributions of these platelet collagen receptors in side-by-side comparisons *in vivo* and *in vitro* [15], [20], [21]. Here we report data comparing GPVI^−/−^, FcRγ^−/−^, and α2β1^−/−^ mice using *in vivo* and *in vitro* assays of thrombosis. Unexpectedly, the GPVI^−/−^ and FcRγ^−/−^ mice demonstrated different defects, suggesting distinct phenotypes of platelets lacking GPVI or FcRγ. These data show that the platelet responses to collagen in FcRγ^−/−^ mice differ from GPVI^−/−^ mice and raises caution to utilizing these two knockout mice as similar systems.

## Materials and Methods

### Materials

Collagen I from rat-tail tendon was purchased from Upstate Cell Signaling Solutions. Bovine serum albumin (BSA), DMSO, glutarahldehyde, EDTA, MgCl_2_, PGE_1_, p-nitrophenol-N-acetyl-β-D-glucosaminide, Apyrase, and other chemicals were purchased from Sigma Aldrich. Anti-phosphotyrosine (p-Tyr-100), anti-phospho-Syk (Tyr 525/526), and anti-RhoGDI (#2564) monoclonal antibodies were purchased from Cell Signaling Technology. Anti-actin polyclonal antibody (C11) was purchased from Santa Cruz Biotechnology. Goat anti-mouse and mouse anti-goat secondary antibodies conjugated to horseradish peroxidase and West-femto chemiluminescence substrate were purchased from Pierce. Hank's Balanced Salt Solution lacking divalent cations (HBSS-) was purchased from Invitrogen.

The α2β1^−/−^ mice, backcrossed onto the C57BL/6 background were previously reported [20]. FcRγ-deficient mice on the C57BL/6 and C57BL/6 X 129/SvJ background were purchased from Jackson Labs. GPVI-deficient mice on a C57BL/6 X 129/SvJ background were developed by Kato *et al*. [15]. GPVI-deficient mice were backcrossed 8 times to the C57BL/6 background using a microsatellite marker-assisted selection (“speed congenics”), as previously described [20]. Animals were housed in pathogen-free conditions at Vanderbilt University Medical Center in compliance with IACUC regulations. The protocol was approved by the Internal Review Board at Vanderbilt University (protocol #M/05/324). All animals were appropriately age and sex matched, and efforts were made to minimize suffering.

### Platelet isolation

Murine PRP or washed platelets were prepared from blood obtained on the day of the experiment according to protocols described previously [22].

### Platelet adhesion assay

Adhesion assays were carried out using washed platelets (1×10^8^ platelets/mL) as done previously [22]. Measurements for each data point were performed in triplicate.

### Platelet aggregation

Aggregation assays using PRP were performed on a BIO/DATA Corporation PAP-4 aggregometer at 37°C with stirring (1200 rpm) as described [20]. Agonists were added at designated final concentrations.

### *In vivo* photochemical injury of the carotid artery of mice

Carotid artery thrombosis was induced as described previously [8]. Briefly, male mice approximately 12 weeks of age were anesthetized with an intraperitoneal injection of sodium pentobarbital, secured in the supine position, and placed under a dissecting microscope. The right common carotid artery was isolated through a midline cervical incision, and an ultrasonic flow probe (Model 0.5 VB; Transonic Systems) was applied. A 1.5-mW, 540-nm laser beam (Melles Griot) was applied to the artery from a distance of 6 cm. Rose-Bengal dye (Fisher Scientific), 50 mg/kg body weight, was then injected into the tail vein, and flow in the vessel was monitored until complete occlusion occurred.

### *In vivo* collagen-induced pulmonary thromboembolism in mice

Collagen-induced thrombosis was carried out as previously described [8]. Briefly, female mice were anesthetized by intraperitoneal injection of 100 to 150 µL of a mixture of ketamine and xylazine. Blood was collected into EDTA-coated microtainer tubes for determination of the baseline platelet count and hematocrit. 25 µg of collagen (equine tendon Type I fibrillar collagen) along with 1 µg epinephrine (Sigma) in phosphate-buffered saline (PBS), or PBS alone, were injected into the right jugular vein; 1 minute after injection a second blood sample was taken and cell counts were measured. Mice were humanely sacrificed 3 minutes after injection and lungs were collected and placed in formalin. Pulmonary thrombi were quantitated using digital imaging of lung sections stained with hematoxylin and eosin using an Olympus Camedia C-3040 Zoom camera. Five random 20X fields were photographed for each specimen. Analysis of thrombus number for each mouse lung was made using Olympus Camedia Master 2.5 software, and then expressed as thrombi per square millimeter±SEM.

### Scanning electron microscopy

Platelet adhesion assays similar to those described above were performed with minor changes. Platelets at a concentration of 2×10^7^ platelets/mL were allowed to adhere to substrates (30 µg/mL) bound to round glass coverslips (Electron Microscopy Sciences; 22 mm diameter) for 1 hour at 37°C. Coverslips were washed 3 times with adhesion buffer. Adherent platelets were fixed using 2% glutaraldehyde for 30 minutes at 21°C, washed 3 times with 0.1 M sodium cacodylate buffer and processed (fixed, dried, and sputter coated) in the VUMC Cell Imaging Shared Resource and the EM Core. Imaging was done using a Hitachi S-4200 Scanning Electron Microscope.

### Mouse platelet phosphotyrosine analysis

Mouse platelets were resuspended at a concentration of 5×10^8^ platelets/mL in HBSS- containing 2 mM MgCl_2_. Mouse platelets (wild type, GPVI^−/−^, or FcRγ^−/−^) were either untreated or stimulated with 10 µg/mL collagen Type I for 1 minute at 21°C after which an excess of ice-cold HBSS- was added to stop the interaction. Platelets were pelleted at 4,000 rpm for 4 minutes at 4°C. Platelets were lysed using SDS-PAGE sample buffer containing protease and phosphatase inhibitors and run on a reducing 10% SDS-PAGE gel. The proteins were transferred to a nitrocellulose membrane followed by immunoblot analysis with anti-phosphotyrosine (1∶2000) or anti-actin (1∶2000) antibodies. Appropriate secondary antibodies linked with horseradish peroxidase were used with a chemiluminescence substrate to image the labeled protein bands using a BioRad ChemiDoc with Quantity One software. Densitometry on protein bands of interest from image files was done using ImageJ software.

### Statistical analyses

The data from multiple different animals were analyzed by Fisher's least significant difference approach. Means, standard deviations (SD), standard error of the means (SEM), t-test, one-way, and two-way ANOVA for column statistics, and nonlinear curve fits were calculated using GraphPad Prism 4 software.

## Results

### Deletion of the GPVI and FcRγ receptors, but not the α2β1 integrin alters collagen-induced intravascular thrombosis and pulmonary embolism

**Figure 1:**
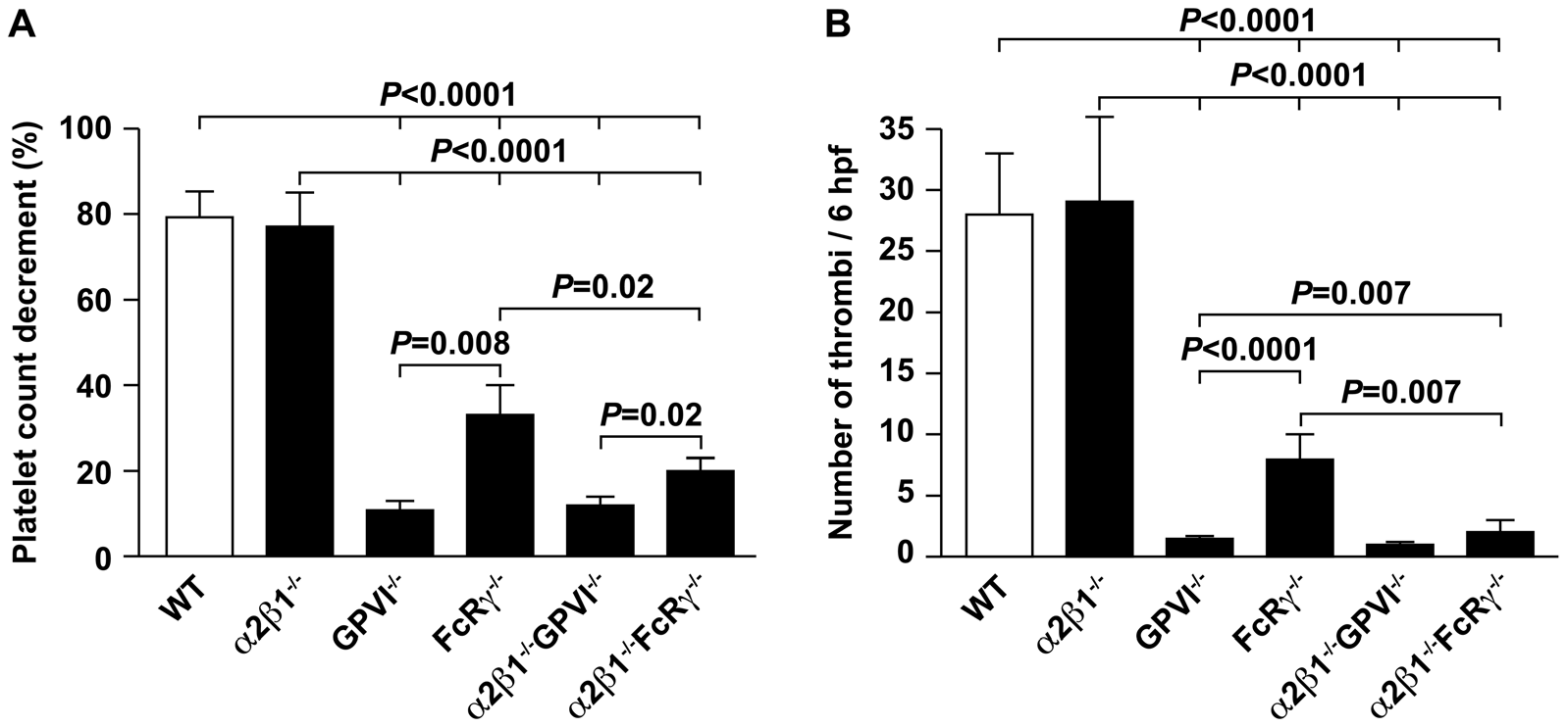
Deletion of the GPVI and FcRγ receptors, but not the α2β1 integrin alters collagen-induced intravascular thrombosis and pulmonary embolism. (**A**) The percentage decrease in platelet count 3 minutes after injection of Type I collagen (25ug) and epinephrine (1ug) was determined for wild type (WT), α2β1^−/−^, GPVI^−/−^, FcRγ^−/−^, α2β1^−/−^/GPVI^−/−^, and α2β1^−/−^/FcRγ^−/−^ mice on the mixed genetic background (129/SvJ × C57BL/6). (**B**) The number of pulmonary thrombi after injection of Type I collagen was determined for wild type (WT), α2β1^−/−^, GPVI^−/−^, FcRγ^−/−^, α2β1^−/−^/GPVI^−/−^, and α2β1^−/−^/FcRγ^−/−^ mice on a 129/SvJ × C57BL/6 background. Thrombi were recorded per mm^2^ in 6 random 20X fields.

Distinct biological roles for the α2β1 integrin and the GPVI-FcRγ receptor in platelet activation and thrombus formation *in vitro* have been well described. To further understand the complex roles played *in vivo* by the α2β1 integrin and the GPVI/FcRγ complex, mice with targeted deletion of the distinct receptors were evaluated using models of collagen-induced thrombosis. Intravenous injection of collagen Type I into wild type animals resulted in rapid onset of intravascular thrombosis, a profound decrease in the number of circulating platelets, massive pulmonary emboli, and death (as previously reported and Figure 1A and B). Pulmonary thrombosis occurred rapidly, therefore the experiment was completed in 3 minutes. It was difficult to evaluate time-dependent differences in this model in which collagen-induced platelet activation is independent of shear stress.

Since the GPVI^−/−^ mice were generated on a mixed 129/SvJ/C57BL/6 background, as previously reported, we first studied the GPVI^−/−^, α2β1^−/−^, FcRγ^−/−^ and littermate control wild type mice on the mixed genetic background. Baseline platelet counts of α2β1^−/−^, GPVI^−/−^, and FcRγ^−/−^ mice were similar to the wild type controls (data not shown). Age-matched animals of all four genotypes were injected with soluble collagen and platelet counts were evaluated after 1 minute. A percentage decrease in platelet count was quantitated using the platelet count before, compared to 1 minute after collagen injection. The platelet count in wild type (n = 13) and α2β1^−/−^ mice (n = 17) decreased by 79.4%±6% and 77.6%±8.2%, respectively. These values were not significantly different and agreed with previously reported data [8]. In contrast, the platelet count in FcRγ^−/−^ and GPVI^−/−^ mice decreased by 38%±5% (n = 7) and 11%±1% (n = 7), respectively (Figure 1A). Platelet count decrement was therefore significantly less in both the FcRγ^−/−^ and GPVI^−/−^ mice when compared to wild type (*P*<0.0001 for both) or α2β1^−/−^ mice (*P*<0.0001 for both). Surprisingly, the platelet count decrease in GPVI^−/−^ animals was also significantly less than the platelet count decrease in FcRγ^−/−^ mice (*P* = 0.008), suggesting a difference in collagen-induced thrombosis between mice lacking the GPVI receptor and the FcRγ chain (Figure 1A).

Quantitative analysis of pulmonary thrombi revealed a large number of intravascular thrombi in wild type and α2β1^−/−^ mice [28±5 thrombi/mm^2^ (n = 13) and 29±7 thrombi/mm^2^ (n = 17) (*P*>0.5)], respectively (Figure 1B). The number of thrombi formed in FcRγ^−/−^ mice [8±2 thrombi/mm^2^ (n = 8)] or GPVI^−/−^ mice [1.5±0.2 thrombi/mm^2^ (n = 8)] was significantly reduced when compared to wild type or α2β1^−/−^ mice (*P*<0.0001 for each analysis) (Figure 1B). Although the FcRγ^−/−^ mice developed significantly fewer thrombi than either the wild type or the α2β1^−/−^ mice, the number of thrombi in the lungs of FcRγ^−/−^ mice was significantly greater than in the GPVI^−/−^ mice (*P*<0.0001). These data further support the difference between GPVI^−/−^ and FcRγ^−/−^ mice in thrombotic response to collagen.

### In the absence of FcRγ receptor, the α2β1 integrin contributes to intravascular thrombus formation

To define the overlapping and/or synergistic roles of the platelet collagen receptors *in vivo*, we compared platelet decrement and pulmonary thrombi in mice with combined deficiency of the GPVI or FcRγ receptor and the α2β1 integrin on a mixed SvJ129/C57BL/6 background. The platelet count decrement in the α2β1^−/−^/FcRγ^−/−^ animals was 20%±3% (n = 8), significantly less than the platelet decrement in wild type or α2β1^−/−^ mice (*P*<0.0001 for both analyses), but surprisingly also significantly less than the decrement in the FcRγ^−/−^ mice (*P* = 0.02) (Figure 1A). In the α2β1^−/−^/GPVI^−/−^ mice (n = 10), the platelet count decreased by 12%±2% (n = 10) (Figure 1A), therefore, significantly less than wild type mice or α2β1^−/−^ mice (*P*<0.0001 for both) and significantly less than α2β1^−/−^/FcRγ^−/−^ (*P* = 0.02) but not different from mice lacking the GPVI receptor alone (*P* = 0.99). The lack of a difference in mice lacking both GPVI and α2β1 integrin may be due to the very low level of platelet aggregation and further changes could not be detected. However, loss of α2β1 did attenuate thrombosis when paired with loss of FcRγ chain.

Thrombus formation in the lungs was evaluated in all genotypes (Figure 1B). The number of thrombi identified in the lungs of α2β1^−/−^/FcRγ^−/−^ animals was 2±1 thrombi/mm^2^ (n = 8) and statistically decreased when compared to the number of thrombi in the lungs of wild type, α2β1^−/−^ (*P*<0.0001 for both), or FcRγ^−/−^ mice (*P* = 0.007). α2β1^−/−^/FcRγ^−/−^ animals developed more thrombi than GPVI^−/−^ mice (*P* = 0.007). Only rare thrombi were identified in the lungs of either α2β1^−/−^/GPVI^−/−^ animals [1±0.2 (n = 10)] or GPVI^−/−^ mice [1.5±0.2 (n = 8)]. These data suggest that in the absence of the FcRγ, the α2β1 integrin plays a significant role in the intravascular thrombi development. In the absence of the GPVI receptor, the response to collagen is so low that an additional contribution by the α2β1 integrin could not be defined.

### Deletion of either the α2β1 integrin or the GPVI subunit, but not the FcRγ receptor delays carotid artery thrombosis

**Figure 2:**
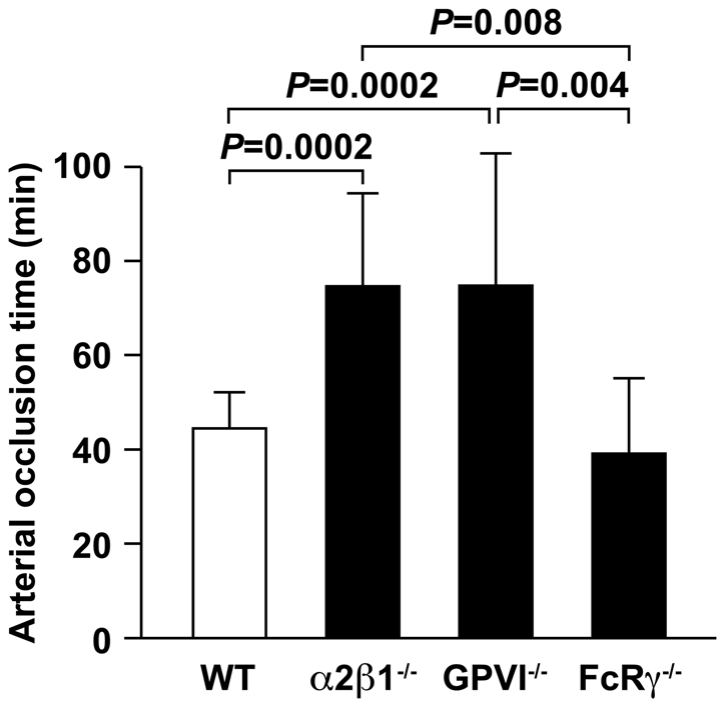
Deletion of either the α2β1 integrin or the GPVI subunit, but not the FcRγ receptor delays carotid artery thrombosis. The length of time to complete arterial occlusion following photochemical injury of the carotid artery was recorded in wild type (WT), α2β1^−/−^, FcRγ^−/−^, and GPVI^−/−^ mice on a mixed genetic background (129/SvJ × C57BL/6). The values represent the mean ± SD for WT (n = 12), α2β1^−/−^ (n = 15), GPVI^−/−^ (n = 7), or FcRγ^−/−^ (n = 7) animals.

We analyzed platelet activation in a second *in vivo* model of thrombosis that involves photochemical damage of the artery to produce endothelial cell denudation and subendothelial extracellular matrix exposure under shear stress conditions [23]. This assay uses laser-activated Rose-Bengal dye to produce the photochemical injury of the mouse carotid artery in order to measure the time to complete vessel occlusion (Figure 2). We compared the time required for complete arterial occlusion in α2β1^−/−^, FcRγ^−/−^, GPVI^−/−^ and wild type mice. As previously reported, the time to complete occlusion was significantly prolonged in the α2β1^−/−^ animals (74.5±19.8 minutes) compared to wild type littermates (44.4±7.8 minutes) (*P* = 0.0002) and to animals lacking FcRγ (*P* = 0.008). Occlusion times for FcRγ^−/−^ mice (39.3±15.9 minutes) were not different from wild type mice (*P* = 0.99). In contrast, occlusion times for the GPVI^−/−^ mice (74.6±28.3 minutes) were statistically increased compared to those in wild type (*P* = 0.0002) and FcRγ^−/−^ mice (*P* = 0.004), but similar to α2β1^−/−^ mice (*P* = 0.3). In this second *in vivo* model of platelet function, the time to complete arterial occlusion was different in the FcRγ^−/−^ and GPVI^−/−^ mice.

### The *in vivo* thrombotic differences are independent of genetic background

**Figure 3:**
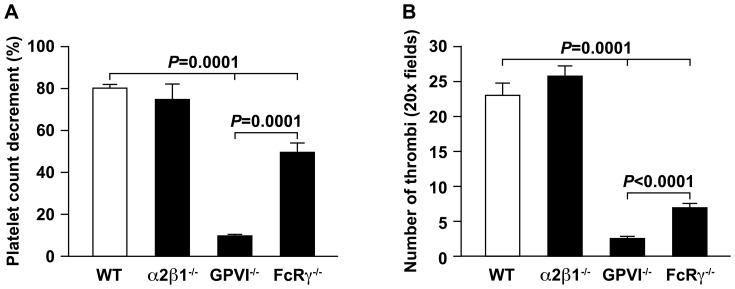
The *in vivo* thrombotic differences are independent of genetic background. (**A**) The platelet count decrement (% change from baseline) following intravenous injection of Type I collagen (25ug) and epinephrine (1ug) into wild type (WT) (n = 7), GPVI^−/−^ (n = 7), FcRγ^−/−^ (n = 9) or α2β1^−/−^ (n = 11) mice on a pure C57BL/6 background was determined. (**B**) The number of thrombi observed in the lungs at 3 minutes after injection of Type I collagen (25ug) and epinephrine (1ug) was determined for wild type (WT), α2β1^−/−^, GPVI^−/−^, FcRγ^−/−^ mice on the C57BL/6 background. Thrombi were recorded per mm^2^ in 6 random 20X fields.

Since the differences observed between the GPVI^−/−^ and FcRγ^−/−^ mice were unexpected and platelet responses are known to be dependent in some circumstances on genetic background, we acquired or generated animals on a pure C57BL/6 background, as described in Methods. Studies of platelet response to collagen were repeated and platelet count decrements in mice on a pure C57BL/6 background were determined. The results obtained for platelet count decrement and number of pulmonary thrombi following intravenous injection of collagen into wild type, GPVI^−/−^, FcRγ^−/−^ or α2β1^−/−^ pure C57BL/6 animals were similar to that observed on the mixed background (Figure 3A and 3B). As observed in mice on a mixed background, there was no difference in platelet count decrement or in the number of thrombi between wild type (n = 7) and α2β1^−/−^ animals (n = 11) (*P* = 0.4), but a significant difference was seen between wild type animals and FcRγ^−/−^ (n = 9) (*P* = 0.0001) or GPVI^−/−^ mice (n = 7) (*P* = 0.0001). In addition, as observed on the mixed background, there was a significant difference in platelet decrement (*P* = 0.0001) and the number of thrombi (*P*<0.0001) between the FcRγ^−/−^ and GPVI^−/−^ mice on the pure background

**Figure 4:**
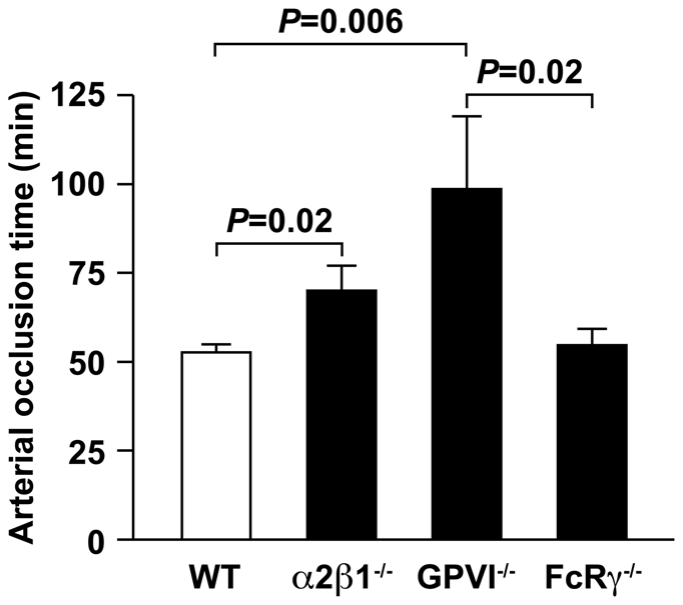
Differences in thrombotic occlusion of the carotid artery are independent of genetic background. The impact of genetic background on photochemically induced endothelial injury on carotid artery occlusion was also evaluated in α2β1^−/−^, FcRγ^−/−^, GPVI^−/−^ and wild type mice on a pure C57BL/6 genetic background. The length of time to complete vessel occlusion following photochemical injury was measured in wild type, α2β1^−/−^, FcRγ^−/−^, and GPVI^−/−^ mice on the pure genetic background (C57BL/6). The values represent the mean ± SD of WT (n = 10), α2β1^−/−^ (n = 10), GPVI^−/−^ (n = 5), or FcRγ^−/−^ (n = 8).

The impact of genetic background on injury induced carotid artery occlusion was also evaluated in α2β1^−/−^, FcRγ^−/−^, GPVI^−/−^ and wild type mice on a pure C57BL/6 genetic background (Figure 4). Time to complete occlusion for the α2β1^−/−^ mice (70±7 minutes) was significantly prolonged compared to wild type animals (52.7±2.2 minutes), as expected (*P* = 0.02). The FcRγ^−/−^ mice demonstrated an occlusion time of 54.8±4.8 minutes, similar to wild type (*P* = 0.7). Time to occlusion for the pure GPVI^−/−^ mice (99±20 minutes) was significantly prolonged compared either wild type (*P* = 0.006) or FcRγ^−/−^ mice (*P* = 0.02). These data show that mice deficient in the GPVI receptor, but not the FcRγ chain manifest a major defect in carotid artery thrombosis induced by photochemical injury. Overall, the genetic background did not contribute to the *in vivo* variances observed between genotypes in thrombosis.

### Platelet adhesion, spreading and aggregation on Type I collagen are dependent on the α2β1 integrin, the GPVI receptor and the FcRγ receptors

**Figure 5:**
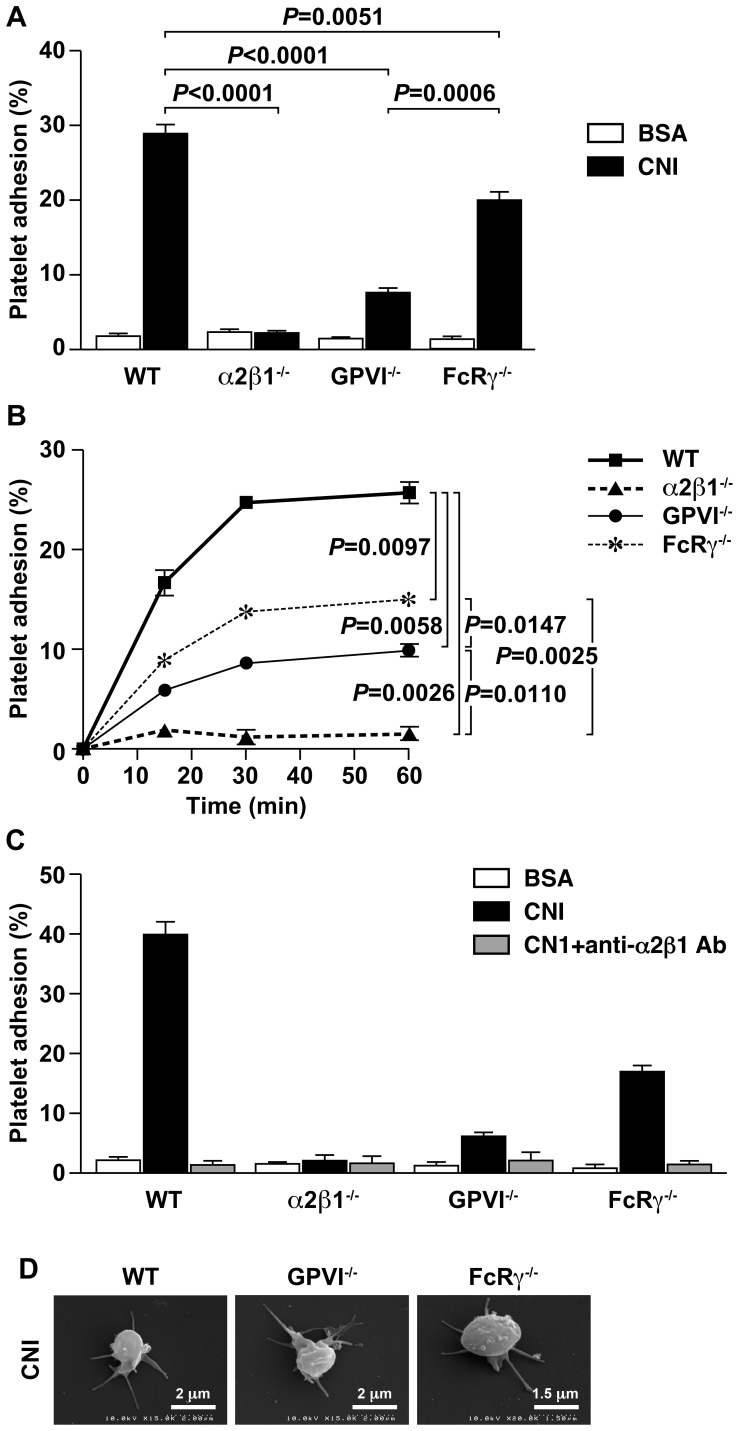
Type I collagen-stimulated platelet adhesion, aggregation, and spreading. (**A** and **B**) Purified platelets isolated from wild type (WT), α2β1^−/−^, GPVI^−/−^, or FcRγ^−/−^ mice were assayed for adhesion to Type I collagen (CNI), or BSA for 60 minutes (**A**) or over a time course of 60 minutes (**B**) *in vitro*. Results represent percentages of adherent platelets (mean of 3 independent experiments performed in triplicate). (**C**) Purified platelets isolated from wild type (WT), α2β1^−/−^, GPVI^−/−^, or FcRγ^−/−^ mice were assayed for adhesion to Type I collagen (CNI) in the presence or absence of inhibitory anti-α2β1^−/−^ antibody or BSA for 60 minutes *in vitro*. Results represent percentages of adherent platelets (mean of 2 independent experiments performed in triplicate). (**D**) Scanning electron micrographs detail wild type (WT), GPVI^−/−^, or FcRγ^−/−^ mouse platelets when adherent to collagen I (CNI) for 60 minutes. Platelets from α2β1^−/−^ mice failed to adhere and therefore were not observed.

The data presented above describe an unexpected difference *in vivo* in thrombosis between mice lacking the GPVI and the FcRγ subunits. To better define the mechanisms for this difference, platelet adhesion using platelets from pure C57BL/6 animals was evaluated *in vitro* at 60 minutes (Figure 5A). Wild type platelets adhered to collagen substrates in a time dependent manner, but not to BSA (Figure 5A). As previously reported, α2β1^−/−^ platelets failed to adhere. Although FcRγ^−/−^ and GPVI^−/−^ platelets adhered to collagen substrates, adhesion was significantly reduced at 60 minutes compared to adhesion of wild type platelets. Although reduced compared to wild type, adhesion of FcRγ^−/−^ platelets was significantly greater than adhesion of GPVI^−/−^ platelets at each time point (Figure 5B). Expression of the α2β1 integrin was similar on GPVI^−/−^ and FcRγ^−/−^ platelets and therefore did not explain the difference in adhesion to collagen I (data not shown).

The increased adhesion to Type I collagen demonstrated by FcRγ^−/−^ platelets, compared to GPVI^−/−^ platelets, suggested one of two possibilities: either the α2β1 integrin on FcRγ^−/−^ platelets was expressed in an activated conformation; or, an alternative collagen binding receptor was mediating adhesion. To differentiate these two possibilities, the ability of inhibitory anti-α2β1 integrin antibody to inhibit platelet adhesion was tested. As shown in Figure 5C, the inhibitory anti-α2β1 integrin antibody completely blocked collagen adhesion by wild type, FcRγ^−/−^ and GPVI^−/−^ platelets. Therefore, α2β1 integrin-dependent activation and collagen adhesion is enhanced in the FcRγ^−/−^ platelets when compared to GPVI^−/−^ platelets.

Next we analyzed platelet aggregation. Wild type platelets formed small and large platelet aggregates during adhesion to collagen I after 60 minutes, but FcRγ^−/−^ and GPVI^−/−^ platelets did not (data not shown). There was no difference in platelet aggregation between the FcRγ^−/−^ and GPVI^−/−^ platelets. Correspondingly, wild type and α2β1^−/−^ platelets aggregated in response to soluble collagen, as measured by turbidometric aggregometry, but neither the FcRγ^−/−^ nor GPVI^−/−^ platelets aggregated (data not shown).

We also examined wild type, α2β1^−/−^, GPVI^−/−^ and FcRγ^−/−^ platelets using scanning electron microscopy to determine if there were variances in platelet morphology during adhesion to collagen I (Figure 5D). Individual wild type, GPVI^−/−^, and FcRγ^−/−^ formed similar filopodial extensions on collagen I. No α2β1^−/−^ platelets attached to collagen I and therefore the image was similar to the BSA negative controls (data not shown).

### Protein phospho-tyrosine analyses of wild type, GPVI^−/−^, and FcRγ^−/−^ mouse platelets

**Figure 6:**
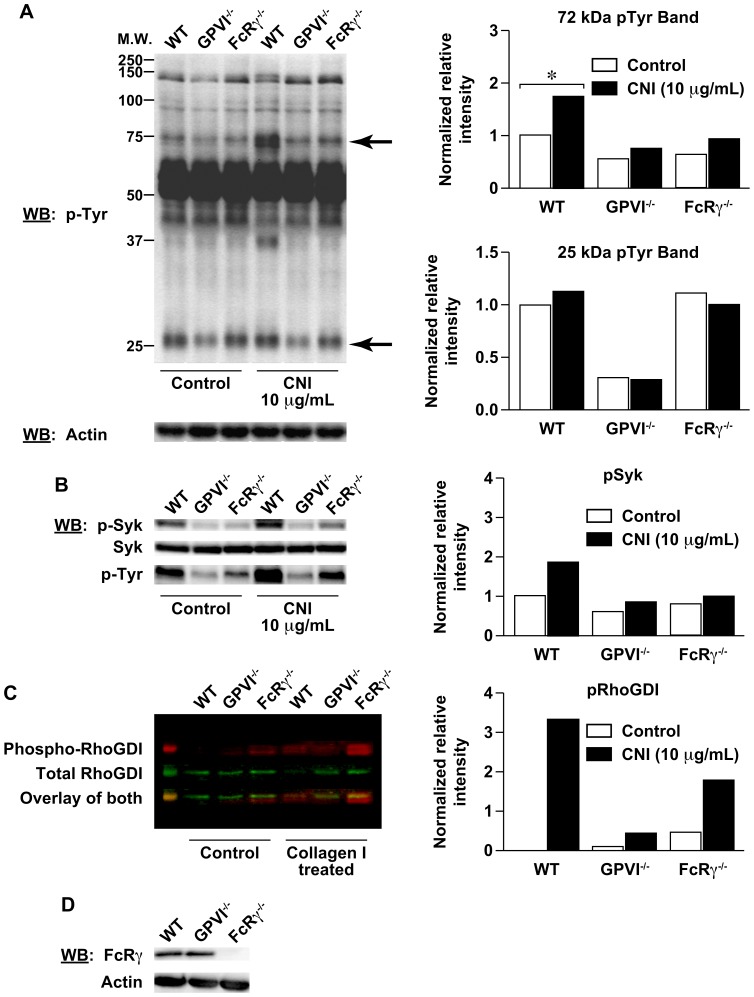
Protein phospho-tyrosine analyses of wild type (WT), GPVI^−/−^, and FcRγ^−/−^ mouse platelets. (**A**) Purified platelets from WT, GPVI^−/−^, or FcRγ^−/−^ mice were either untreated or stimulated with 10 µg/mL collagen I In the presence of 2 mM MgCl_2_ for 1 minute followed by immunoblot analysis using antibodies against phospho-tyrosine (pTyr) or actin. Protein bands of interest are indicated with arrows at molecular weights of about 25 and 72 kDa. Quantification of the 25 and 72 kDa pTyr bands were performed by densitometry and normalized to actin. (**B**) WT, GPVI^−/−^, or FcRγ^−/−^ mouse platelets stimulated with CNI were analyzed for phosphorylated Syk (pSyk) and total Syk by immunoblot. In the presence of 2 mM MgCl_2_, control platelets or platelets treated with 10 µg/mL CNI for 1 minute, were lysed, and analyzed by Western blot using antibodies for phospho-Syk (Tyr525/526), total Syk protein, or total phospho-tyrosine. Quantification of the pSyk and total Syk was performed by densitometry and the value of pSyk normalized to total Syk. (**C**) Western blot analysis of WT, GPVI^−/−^, or FcRγ^−/−^ mouse platelets for phosphorylated RhoGDI and total RhoGDI following CNI stimulation as described. Quantification of the pRhoGDI and total RhoGDI was performed and the value of pRhoGDI normalized to total RhoGDI. (**D**) Western blot analysis of unstimulated WT, GPVI^−/−^, or FcRγ^−/−^ mouse platelets for total FcRγ protein or actin expression.

To determine whether the different phenotypes observed *in vivo* between wild type, GPVI^−/−^, and FcRγ^−/−^ mice were a consequence of alterations in α2β1 integrin-dependent platelet activation signals in response to collagen, we determined the extent of protein tyrosine phosphorylation following collagen-induced platelet activation *in vitro* (Figure 6A). Mouse platelets from wild type, GPVI^−/−^, or FcRγ^−/−^ mice were either untreated or stimulated with 10 µg/mL collagen I for 1 minute followed by immunoblot analysis of proteins containing phosphorylated tyrosines. Interestingly, control and collagen-stimulated platelets from wild type, GPVI^−/−^, and FcRγ^−/−^ animals showed slightly or moderately different levels of tyrosine phosphorylation of 72 and 25 kDa proteins, respectively. These variations in protein tyrosine phosphorylation between genotypes was quantified for the 72 and 25 kDa proteins in Figure 6A, and the amounts of phosphorylation correlates with the levels of thrombosis observed *in vivo* and with adhesion to collagen substrates *in vitro*. Wild type platelets demonstrated a low basal level of phosphorylation of a 72 kDa protein that was augmented by collagen I stimulation. GPVI^−/−^ platelets showed the lowest level of basal and collagen-stimulated tyrosine phosphorylation of the 72 kDa protein. The FcRγ^−/−^ platelets showed intermediate levels of basal and slight but not reproducible level of collagen-stimulated tyrosine phosphorylation of the 72 kDa protein. Both wild type and FcRγ^−/−^ platelets had similar low levels of basal phosphorylation of the 25 kDa protein that were much higher than the levels observed in GPVI^−/−^ platelets. Both wild type and FcRγ^−/−^ platelets demonstrated enhanced phosphorylation of the 25 kDa protein with collagen I stimulation. These data further support an underlying difference between platelet activities in FcRγ^−/−^ and GPVI^−/−^ mice.

A potential candidate for the tyrosine-phosphorylated 72 kDa protein is Syk, a protein tyrosine kinase important in GPVI/FcRγ signal transduction and platelet activation. Therefore, we analyzed Syk phosphorylation at tyrosines 525 and 526, which are required for Syk activation. Mouse platelets (wild type, GPVI^−/−^, or FcRγ^−/−^) were left untreated or stimulated with 10 µg/mL collagen I for 1 minute followed by immunoblot analysis of phospho-Syk (Tyr525/526) in correlation with total Syk protein and total protein tyrosine phosphorylation of the 72 kDa band (Figure 6B). Collagen-treated platelets showed a slightly elevated levels of phospho-Syk (Tyr525/527) that correlated with total phospho-tyrosine of the 72 kDa protein band when comparing wild type, GPVI^−/−^, and FcRγ^−/−^ platelets. Wild type had a low basal level of Syk phosphorylation that increased with collagen I treatment; GPVI^−/−^ had the lowest level of basal and collagen-stimulated Syk phosphorylation; and FcRγ^−/−^ platelets also had low to intermediate levels of basal and collagen-stimulated Syk phosphorylation as shown in the quantitation of phospho-Syk (Figure 6B). The difference in phospho-Syk levels between resting and activated platelets was not significant and therefore did not contribute to observed phenotype.

Identification of a candidate for the 25 kDa phosphotyrosine protein band was undertaken, and a potential candidate of this molecular weight was the family of RhoGDI proteins. RhoGDI has been shown to modulate cellular activities through regulation of Rho GTPases by inhibiting Rho GTPase activation through a sequestration mechanism, which is diminished when RhoGDI in tyrosine phosphorylated [24]. We compared baseline and collagen I-induced RhoGDI2 tyrosine phosphorylation in wild type, FcRγ^−/−^, and GPVI^−/−^ platelets. As shown in Figures 6C, the 25 kDa phospho-protein band overlays with RhoGDI2. Significantly enhanced tyrosine phosphorylation of RhoGDI2 in wild type and FcRγ^−/−^, but not GPVI^−/−^ platelets occurred in response to Type I collagen.

Since α2β1 integrin-dependent collagen adhesion and RhoGDI2 phosphorylation occurred in FcRγ^−/−^ platelets, but not in GPVI^−/−^ platelets, the continued expression of FcRγ in GPVI^−/−^ platelets could be causing a dominant negative phenotype in the GPVI^−/−^ mice by interfering with signaling pathways in these platelets. We therefore determined the expression of FcRγ receptor in platelets from GPVI^−/−^ mice in comparison to wild type and FcRγ^−/−^ (Figure 6D). Surprisingly, GPVI^−/−^ platelets have a similar expression level of FcRγ compared to wild type platelets and not a partial reduction. As expected, no expression was seen in the FcRγ^−/−^ platelets. The wild type expression levels of FcRγ receptor in GPVI^−/−^ platelets may cause the varied thrombotic phenotypes observed between GPVI^−/−^ and FcRγ^−/−^ mice through increased RhoGDI sequestration of Rho GTPases and attenuation on actin cytoskeletal dynamics, which are important in platelet adhesion and thrombosis.

## Discussion

The findings reported in this study demonstrate novel requirements *in vivo* and *in vitro* for the platelet collagen receptors, the α2β1 integrin and the GPVI/FcRγ receptor complex. First, the *in vivo* data demonstrate by using multiple genetically modified animals that deletion of the GPVI receptor results in a phenotype that is distinctly different from that resulting from deletion of the common FcRγ chain. Second, both the α2β1 integrin and the GPVI receptor, but not the FcRγ subunit, influence carotid artery occlusion *in vivo*. In contrast, the loss of GPVI receptor or the FcRγ chain decreases intravascular thrombosis in response to soluble Type I collagen, but surprisingly the FcRγ^−/−^ animals retain some thrombotic potential to collagen. These findings are the first to show disparate effects in thrombosis between GPVI receptor and FcRγ chain knockout mice. Much of our earlier understanding of the role of the GPVI receptor in collagen-induced platelet activation and thrombus formation was based on mice lacking the FcRγ chain. The development of the α2β1 integrin-deficient mice [20], [21], FcRγ-deficient mice, and GPVI-deficient mice [15], affords an unambiguous opportunity to address definitively the roles and contributions of these receptors to platelet activation by collagen *in vivo* and *in vitro*. Third, earlier data from a number of laboratories, including our own, failed to identify a role for the α2β1 integrin in collagen-induced intravascular thrombosis. We now show that in the absence of the common FcRγ chain the α2β1 integrin promotes a low level of collagen-induced platelet activation and thrombosis. Finally, the molecular basis for the difference in activation status of platelets from FcRγ chain-deficient mice and GPVI-deficient mice correlates to differences in collagen-induced tyrosine phosphorylation of RhoGDI2.

The identification of different thrombotic responses to collagen observed in mice with deletion of GPVI or FcRγ genes was initially surprising, and it was logical to assume that this was attributable to the genetic background of mice on a mixed genetic background. A study by Cheli *et al.* [25] using the same mixed GPVI^−/−^ mice on the 129/SvJ x C57BL/6 background identified a *Modifier of hemostasis* (*Mh*) locus on chromosome 4 that correlated with the extreme but transient dichotomy in tail bleeding time (tBT) seen in the earlier generations of these mice. A modest correlation was also observed in the *in vivo* ferric chloride-induced carotid artery injury model. With progressive backcrosses to C57BL/6, this phenotypic difference gradually diminished until later generations of GPVI^−/−^ mice that were congenic on the C57BL/6 background exhibit a normal tBT. Nonetheless, a follow-up study of *Mh* has identified at least one candidate gene of interest that is currently under further investigation. Our studies in this report rule out the possibility that genetic differences arising from the original mixed background (129/SvJ × C57BL/6) are responsible for this unexpected result since animals with genotypes on pure inbred background (C57BL/6) showed similar experimental outcomes suggesting that a genetic modifier (such as *Mh*) was not responsible for the differences. Thus, we conclude that there is yet another mechanism responsible for thrombotic differences between GPVI^−/−^ or FcRγ^−/−^ mice.

To determine whether differences observed *in vivo* were demonstrable in isolated platelets, the ability of FcRγ^−/−^ and GPVI^−/−^ platelets to respond to collagen was determined. GPVI^−/−^ and FcRγ^−/−^ platelets adhered to Type I collagen substrates, however FcRγ^−/−^ platelets were significantly more adherent to collagen in an α2β1 integrin-dependent manner than GPVI^−/−^ platelets. No differences between GPVI^−/−^ and FcRγ^−/−^ platelets were seen in *in vitro* analyses of aggregation or morphology. Taken together these results suggested that FcRγ^−/−^ platelets, but not GPVI^−/−^ platelets, adhered to collagen in an α2β1 integrin-dependent fashion. During the platelet response to collagen, distinct phospho-tyrosine protein profiles were observed between the GPVI^−/−^ and FcRγ^−/−^ platelets, especially proteins of 72 and 25 kDa. The 25 kDa phosphoprotein was identified as RhoGDI2, an important regulator of RhoGTPase signaling.

Importantly, tyrosine phosphorylation of RhoGDI inhibits binding to RhoA, Rac1, and Cdc42 freeing them for activation [26], [27]. Not much is known about RhoGDI functions in platelets even though its activity on Cdc42 was revealed in platelets [28]–[31]. We report that RhoGDI2 phosphorylation is enhanced in FcRγ^−/−^ platelets when compared to GPVI^−/−^ platelets upon collagen stimulation. This suggests that FcRγ^−/−^ platelets may contain a signaling environment more primed for RhoGTPase activation than GPVI^−/−^ platelets. In agreement with our result, Poole *et al.* observed a slight increase in Syk phosphorylation in FcRγ^−/−^ platelets with collagen I treatment [32]. Mazzucato *et al*. showed that platelets lacking GPVI under flow conditions could still adhere to collagen I and elevate intracellular Ca^2+^ through α2β1 integrin [33]. Awareness of this difference between GPVI/FcRγ knockouts may be pertinent to future research since a recent study by Boulaftali *et al.* showed that ITAM containing receptors (GPVI/FcRγ and CLEC2) were critical for a novel form of hemostasis at sites of inflammation where GPCR signaling was not required [34], [35]. On the basis of these results, we propose that GPVI^−/−^ and FcRγ^−/−^ platelets are poised at different resting states and GPVI^−/−^ platelets have a larger activation barrier to overcome than wild type or FcRγ^−/−^, which explains the differences observed with the *in vivo* thrombotic analyses.

Animals deficient in the common FcRγ chain, an important signaling subunit of multiple cell surface receptors, have been extensively evaluated as a model of GPVI deficiency [32], [36]. These mice, which lack the GPVI/FcRγ receptor, also lack FcεRγI, FcγRIII, and FcγRI, and are immunodeficient with abnormalities in macrophage, NK cell, mast cell and B cell function, but fail to manifest a bleeding diathesis [37]. Abnormalities observed with FcRγ-deficient platelets *in vitro* include defective secretion, platelet activation and aggregation in response to collagen or GPVI-mimetics. Interestingly, bleeding and platelet function abnormalities are much less severe in FcRγ-null mice than in mice lacking several of the downstream adaptor/signaling molecules such as SLP76 or PLCγ2 [38], [39]. These studies initially suggested that other collagen receptors in addition to GPVI/FcRγ also contribute to collagen-induced signals. Work by Konstantinides *et al.* showed that during arterial thrombosis loss of GPVI had variable effects and was dependent upon wound severity and the presence of activating factors [40]. We now raise the possibility that the platelet response to collagen in mice lacking the FcRγ chain differ in a number of ways from mice lacking only the GPVI subunit. To exclude the possibility that expression of the FcRγ receptor on other cell types contributes to the phenotype will require additional experiments with platelet-selective, FcRγ-null animals.

Enhanced adhesion to collagen I and the higher level of protein tyrosine phosphorylation in collagen-stimulated FcRγ^−/−^ platelets compared to GPVI^−/−^ platelets suggests FcRγ expression in GPVI^−/−^ platelets mediates a dominant-negative phenotype. The level of FcRγ expression remains at wild type levels even though there is loss of the GPVI/FcRγ receptor complex, which suggests that FcRγ could be associating with other interacting proteins in GPVI^−/−^ platelets and negatively affecting platelet ITAM signaling pathways through sequestration or disruption mechanisms. Importantly, in mouse platelets there are no other known membrane proteins that directly bind FcRγ. Dominant negative mutations have been shown to affect surface receptor function previously (e.g. PDGF and Her2 receptors) [41], [42]. This inhibitory activity is not present upon loss of FcRγ expression or correct partnering of FcRγ proteins. We speculate that the decreased levels of tyrosine phosphorylated proteins (RhoGDI) in collagen-stimulated GPVI^−/−^ platelets attenuates platelet activities like cytoskeletal rearrangement, priming of α2β1 integrin, or synergy with GPCR signaling, which are important processes of inactivation of wild type platelets [22], [43], [44]. Interestingly, the loss of α2β1 integrin on FcRγ^−/−^ platelets decreased collagen-stimulated aggregation *in vivo* and suggests α2β1 signaling is negatively regulated by inappropriate expression of FcRγ in GPVI^−/−^ platelets.

In summary, we identify *in vivo* and *in vitro* differences in thrombosis between α2β1^−/−^, GPVI^−/−^ and FcRγ^−/−^ platelets, which surprisingly revealed variances between GPVI^−/−^ and FcRγ^−/−^ platelets. This variation in phenotype seems to be attributable to normal expression levels of FcRγ in GPVI^−/−^ platelets and produces a dominant negative effect through a decrease in protein tyrosine phosphorylation of RhoGDI leading to increased inhibition of RhoGTPases. In addition, these findings suggest a complex signaling network downstream of the platelet collagen receptors. Shida and colleagues recently published a comprehensive analysis of von Willebrand factor and its interactions with collagen in conjunction with GPVI and the α2β1 integrin during thrombosis and showed major but overlapping functions [45]. Further understanding of the functions of GPVI/FcRγ on platelets and the involvement of RhoGDI and other molecules, including von Willebrand factor in this complex pathway is necessary and important as these receptors are potential targets for antithrombotic therapy.
